# 

**Published:** 1887-12

**Authors:** 


### CONTENTS, DEC. 1887, (VOL. III, NO. V )

ORIGINAL COMMUNICATIONS.—A Case of Uterine Pregnancy: Gastrotomy : by W. M. Cunningham, M. D., Bastrop.. 205
Gastrotomy for Incised Wound of Stomach : by J. A. Black, M. D.. Round Rock........................... 213
Remarks on Hæmaturia Miasmatica : by A. N. Perkins, M. D., Sabine Pass.............................. 216
Eye Diseases and other Reflexes, due to Errors of Refraction : by T. J. Tyner, M. D., Austin.................. 218
SOCIETY NOTES.—Annual Meeting West Texas Medical Society; East Line Medical Society: The East Texas Medical Society...................................... 222
Austin District Medical Society, Second quarterly meeting 223
CORRESPONDENCE.—Letter from Dr. C. H. Hughes, on Electricity in Medicine.................................. 230
FOREIGN CORRESPONDENCE.—Letter from Our Paris Correspondent........................................ 233
EDITORIAL.—Shall the State Medical Association Have A Fixed Abode, and Place of Meeting ; A State Medical Library........................................ 239
The County Medical Societies of Texas................ 244
Patronize Home Industry............................ 244
MEDICAL NEWS AND MISCELLANY.—Married : Drs. Maxwell, Cocke, Stone and King............................. 245–6
"Doc" W. M. Yandell : Dr. W. M. Burger............ 246
Dr. F. A. Maxwell: Physicians' Mutual Benefit Association of Texas : Important to Medical Colleges : Cocaine in Gout : U. S. Pension Examiner—Dr. O. Eastland, Dr. W. H. Wilkes, Dr. J. T. Musick, North Texas Medical Society, Dr. J. L. Felder, Dr. F. E. Yoakum... 249
ADVERTISERS NOTICES................................ 250

